# Urine as a Source of Liquid Biopsy for Cancer

**DOI:** 10.3390/cancers13112652

**Published:** 2021-05-28

**Authors:** Masanori Oshi, Vijayashree Murthy, Hideo Takahashi, Michelle Huyser, Maiko Okano, Yoshihisa Tokumaru, Omar M. Rashid, Ryusei Matsuyama, Itaru Endo, Kazuaki Takabe

**Affiliations:** 1 Breast Surgery, Department of Surgical Oncology, Roswell Park Comprehensive Cancer Center, Buffalo, NY 14263, USA; Masanori.Oshi@RoswellPark.org (M.O.); vijayashree.murthy@roswellpark.org (V.M.); Michelle.Huyser@RoswellPark.org (M.H.); Yoshihisa.Tokumaru@RoswellPark.org (Y.T.); 2 Department of Gastroenterological Surgery, Yokohama City University School of Medicine, Yokohama 236-0004, Japan; ryusei@yokohama-cu.ac.jp (R.M.); endoit@yokohama-cu.ac.jp (I.E.); 3 Hepatopancreatobiliary (HPB), Surgical Oncology, Department of Surgery, Mount Sinai South Nassau, One South Central Avenue, Valley Stream, NY 11580, USA; hideo.takahashi@snch.org; 4 Department of Breast Surgery, Fukushima Medical University, Fukushima 960-1295, Japan; tentekomaikocco@hotmail.com; 5 Department of Surgical Oncology, Graduate School of Medicine, Gifu University, 1-1 Yanagido, Gifu 501-1194, Japan; 6 Michael and Dianne Bienes Comprehensive Cancer Center, Department of Surgery, Holy Cross Hospital, Fort Lauderdale, FL 33308, USA; omarmrashidmdjd@gmail.com; 7 Department of Surgery, Massachusetts General Hospital, Boston, MA 02114, USA; 8 Department of Surgery, University at Buffalo Jacobs School of Medicine and Biomedical Sciences, The State University of New York, Buffalo, NY 14263, USA; 9 Department of Breast Surgery and Oncology, Tokyo Medical University, Tokyo 160-8402, Japan; 10 Department of Surgery, Graduate School of Medical and Dental Sciences, Niigata University, Niigata 951-8510, Japan

**Keywords:** liquid biopsy, urine, urine liquid biopsy, DNA, mRNA, microRNA, sncRNA

## Abstract

**Simple Summary:**

Tissue biopsy is essential for diagnosis and characterization of a tumor. Recently circulating tumor cells and other tumor-derived nucleic acid can be detected from blood, which is called liquid biopsy. Now this concept has been expanded to many other body fluids including urine. Urine is the least invasive method to obtain a liquid biopsy and can be done anywhere, which allows longitudinal repeated sampling. Here, we review the latest update on urine liquid biopsy in urological and non-urological cancers.

**Abstract:**

Tissue biopsy is the gold standard for diagnosis and morphological and immunohistochemical analyses to characterize cancer. However, tissue biopsy usually requires an invasive procedure, and it can be challenging depending on the condition of the patient and the location of the tumor. Even liquid biopsy analysis of body fluids such as blood, saliva, gastric juice, sweat, tears and cerebrospinal fluid may require invasive procedures to obtain samples. Liquid biopsy can be applied to circulating tumor cells (CTCs) or nucleic acids (NAs) in blood. Recently, urine has gained popularity due to its less invasive sampling, ability to easily repeat samples, and ability to follow tumor evolution in real-time, making it a powerful tool for diagnosis and treatment monitoring in cancer patients. With the development and advancements in extraction methods of urinary substances, urinary NAs have been found to be closely related to carcinogenesis, metastasis, and therapeutic response, not only in urological cancers but also in non-urological cancers. This review mainly highlights the components of urine liquid biopsy and their utility and limitations in oncology, especially in non-urological cancers.

## 1. Introduction

Radiological evaluation followed by biopsy for assessment of tumor tissue and pathological confirmation are the main investigatory methods for cancer diagnosis and treatment planning. Depending upon the location of the tumor, invasive biopsies can be painful with risk of complication and associated costs. This is particularly the case when lesions are in vital organs or close to major vessels, thus making biopsy very challenging to access. Treatment decisions are often made based on the pathological profile of the primary tumors, which may or may not be the same genomic clone of the metastatic tumor. It is well known that treatment effect is different between primary and metastatic tumors [1]. Cancer cells proliferate continuously, through clonal evolution, to adapt to new environments and exhibit clonal selection by selective pressure from a tumor microenvironment (TME) or treatment [2]. It is now known that a bulk tumor may consist of multiple clones of the same cancer cells (with different molecular and phenotypical profiles) that have a different cancer biology and clonal evolution in response to treatment, also known as intratumor heterogeneity. Intratumor heterogeneity is a key challenge in cancer treatment, requiring real-time assessment of tumor genomic information for precision medicine. Tissue biopsy often takes samples from only a small part of a bulk tumor and thus may not capture the entire spatial diversity of tumor heterogeneity [3]. Although multi-region sequential biopsy can be performed in order to address intratumor heterogeneity [4,5], it may be impractical in clinical practice and limited to the number of samples that can be tolerated by the patient. At the present time, cancer surveillance and assessment of treatment effect is dependent on imaging studies. However, they can only capture morphology of the tumor as a snapshot at a specific time and location, which does not correspond to the whole characteristics or function of the tumor. Multiple follow-up visits with imaging studies and possible biopsies significantly reduce patient compliance and quality of life, and it may be cost-prohibitive. In order to avoid the shortcomings of current imaging and tissue biopsy modalities but capture tumor heterogeneity, a non-invasive method to monitor tumor-wide genomic information during tumor progression or treatment responses is needed.

Liquid biopsy can be an answer to these challenges. Body fluids contain large amounts of substances secreted from cells after they are utilized in intercellular communication or released upon cell death. They include metabolites, proteins, and nucleic acids which may reflect the changes or abnormalities of cells in the body. Liquid biopsy refers to a process of obtaining tumor-derived materials from body fluids. It is a non-invasive investigative modality suited for repetitive assessment of tumor-related substances for assessing changes in gene expression patterns and to study the genomic profile of the tumor. Liquid biopsy (regardless of blood or urine) measures cells or nucleic acids, either secreted out of the tumor or brushed out of the tumor after being destroyed. Thus, liquid biopsy involves sampling from the entire tumor and not a specific area of a bulk tumor. First, in regard to tumor heterogeneity, blood or urine are expected to contain materials secreted or released from all cells and its quantity is expected to be reflective of the amount in the bulk of a tumor. Second, since blood or urine capture the secretome from cells, it is expected to capture the function of the cells. Changes in circulating materials reflect overall changes in the TME, such as stromal interactions between the cancer and immune cells [6]. Theoretically, liquid biopsy has the possibility to capture everything from the cells in the TME and is not spatially or longitudinally limited. Finally, based upon the homeostasis of the body, anything produced and secreted by a neoplasm should be an excess to the body and should be excreted via the urine; thus, urine is theoretically an ideal medium to detect a neoplasm-derived material.

Because of these advantages, liquid biopsy is expected to become a powerful tool in oncology not only for diagnosis and prognosis, but also for surveillance and assessing therapeutic effects. Liquid biopsy initially referred only to circulating tumor cells (CTCs) (although with a short half-life) but now extends to other components released by tumors like cell-free circulating nucleic acids (NAs) such as DNA, messenger RNA (mRNA), microRNA (miRNA), non-coding RNA, extracellular vesicles (EVs or exosomes) and tumor educated platelets (TEPs). Liquid biopsy corresponds to tumor burden and measurement of ctDNA appears to be even more beneficial in the metastatic setting (with levels < 5 CTCs per 7.5 cc correlating to better progression free survival and overall survival) [7]. For the same reason, high false negative rates (FNR) are seen when lower levels of tumor-derived products are seen in body fluids. For example, cell-free DNA (cfDNA) can be poor in quality secondary to inflammation or infections that result in high false positive rates. Droplet digital polymerase chain reaction (ddPCR) has become one of the most sensitive methods for detection of somatic mutations by improving cfDNA extraction methods, thereby optimizing the yield of cfDNA [8,9,10,11]. Although liquid biopsy can be performed with various body fluids, such as blood [12], CSF [13,14], pleural fluid [15,16], gastric juice [17,18], or saliva [19,20], we will focus on urine, as it can be easily and non-invasively collected without use of any special techniques or instruments and in copious amounts. Table 1 illustrates the specifics of standard tissue biopsy and its comparison with blood and urine as liquid biopsy. While several reviews have been published on urine as a source of liquid biopsy for cancer, most of them have mainly focused on genitourinary cancers. Chen et al. focused on urine liquid biopsy technologies and its use in cancer, glomerular disease, and tuberculosis [21], while Yu et al. focused on prostate and bladder cancer [22], and Hentschel et al. on bladder, prostate, and cervix cancer [23]. This review seeks to highlight the components of urine liquid biopsy and its utility and limitations in oncology, mainly in non-urological cancers.

## 2. Urine Liquid Biopsy Components

Urine is a biological fluid consisting of organic and inorganic compounds; salts; cells like leucocytes, renal cells, urothelial cells, prostate cells, and exfoliated tumor cells; and tumor cell-free nucleic acids. Tumor-derived DNA, mRNA, and miRNA can be obtained via whole urine sample, centrifugation to obtain urine sediment, or filtration to obtain urinary supernatant and cells [24]. With recent technological advances, it has become possible to extract and analyze minute amounts of NAs from body fluids. It is easy to collect large amounts of urine for larger samples of urinary NAs. Urinary NAs are expected to provide very useful clinical information that may reflect tumor heterogeneity.

### 2.1. Urinary DNAs

While the exact mechanism of origin of circulating tumor DNA (ctDNA) and its filtration by the kidneys remains unclear, some hypotheses of origin include: (i) from dying cells, exfoliated either in urine (bladder and prostatic cells) or from circulation (ii) from CTCs and (iii) via active release [25]. Urine contains DNA as a result of renal clearance of blood. Only molecular substances smaller than 6.4 nm in diameter and molecular weight not greater than 70 kDa can pass through the lumen of a nephron [26]. This corresponds to about 100 base pair (bp) DNA in size [27]. Since the size of a mononucleosome exceeds the size of the nephron barrier pores, they cannot pass through the nephron. Only protein and NAs can pass through and are excreted in the urine. Many studies of urine liquid biopsy have reported the correlation between urinary DNAs and urological malignancies, such as cancers of the bladder [28,29], prostate [30], and kidney [31], as a result of directly shedding breakdown products in the urine. Isolating DNA fragments in urine is technically easier than blood since urine contains less protein [32]. On the other hand, NA-hydrolyzing enzymes that breakdown DNAs are easily activated in urine. DNA hydrolase deoxyribonuclease I and II (DNase I and II) are present both in urine and blood and are more active in urine. DNase I is a major DNA hydrolase released from the pancreas. The amount of DNase II is less than DNase I in urine although it is more potent.

DNA methylation changes, which are considered one of the primary events in carcinogenesis, can be identified by DNA-sodium bisulfite in the urine. This method selectively deaminates unmethylated cytosines to uracil but methylated forms of cytosines escape the bisulfite reaction, allowing them to be analyzed by polymerase chain reaction (PCR)-based technology to target specific functional locations like CpG islands where methylation genes are expressed. However, there is great variability in its sensitivity and specificity. GSTP1 methylation is a biomarker for prostate cancer [33] and ONECUT2 (One Cut Homeobox 2) is for upper ureteral carcinoma [34]. However, due to its low sensitivity, DNA methylation is recommended only in combination with other biomarkers.

Urinary cell-free DNA (ucfDNA) originates directly from dying cells exfoliated in urine and gives important information regarding DNA derived from cancer cells and is considered to be more representative than the tissue biopsy of a tumor [35]. There are no standard protocols for isolation or detection of ucfDNA to date, but it can be detected by conventional PCR-based assays or by using commercially available kits [36]. Recently, next generation sequencing (NGS) has been used for better sensitivity [37]. ucfDNA has mainly seen utility in urological cancers and was first described by Sidransky et al. in 1991 with the presence of p53 mutations in the urine sediment of patients with muscle invasive bladder cancer [38]. ucfDNA has also been investigated for EGFR mutation in non-small cell lung cancer [39], with elevated levels seen in Stage III and IV. Elevated levels of ucfDNA p53 mutations have been demonstrated by Lin et al. in hepatocellular carcinoma and could be potentially explored for screening [40]. Su et al. reported that KRAS mutation was detected in higher incidence in urine compared to serum (35%) or plasma (40%) among patients with colorectal cancer or colonic polyps [41]. KRAS gene G12/13 mutation has also been found in ucfDNA by the NGS approach of patients with colorectal cancer [42].

### 2.2. RNA-Based Biomarkers

Several types of RNA are present and measurable in the supernatant of the urine. RNA molecules are biochemically unstable and sensitive to heavy metal ions, alkaline pH, and RNA-hydrolyzing enzymes. There are abundant RNA hydrolases in urine, such as RNA-hydrolyzing enzyme (RNase II) and Ribonuclease I, which hydrolyze both RNAs and DNAs. Despite this mechanism, mRNAs are still detectable in the urine because they are somewhat protected from degradation by extracellular vesicles, ribonucleoproteins, and lipoproteins [43]. Through alternate splicing of mRNA, many genes generate different isoforms of protein products in cancer. Thus, mRNA, being a protein coding transcript, represents a good biomarker for establishing the correlation between information in DNA and proteins. Several methods to isolate urinary mRNA have been described, including the QIAamp Circulating Nucleic acid kit (Qiagen) [44], RNeasy kit (Qiagen [45]), and Quick-RNA MicroPrep Kit (Zymo Research) [46], and miRNeasy kit (Qiagen) [47]. After isolation of mRNA, molecular biology methods such as quantitative-PCR, droplet digital PCR, or Next Generation Sequencing are required to search or determine NAs. Currently, the Xpert BC Monitor test [48] and 2-Gene mRNA Urine test [49] are used for bladder cancer and prostate cancer, respectively. Since these kits have been used predominantly for urological cancer, further studies are needed to expand their application for non-urological cancers.

#### 2.2.1. Urinary microRNAs

Compared with mRNA that can be easily degraded by RNA-hydrolyzing enzymes, microRNA is more resistant to nucleases and remains relatively stable in urine [50]. MicroRNA are a class of short single strand RNAs (22–24 nucleotides in length) and are involved in cell proliferation, differentiation, stress response, inflammation, and cell death [51,52,53,54,55,56,57,58]. They epigenetically inhibit the translation of target mRNA into proteins [59]. They are known to play roles in different mechanisms of cancer progression, including carcinogenesis, angiogenesis, and metastasis [51]. MicroRNA is encapsulated and bound to RNA-binding protein, which stabilize it to the point that it withstands several cycles of freeze and thaw and remains stable at room temperature for long periods of time. They can be evaluated in different fractions such as non-centrifuged urine, urine sediment, supernatant, and as part of exosomes [60]. Since some microRNAs released from cancer cells are also highly expressed in activated T-cells, some suggest that monitoring circulating microRNA released from the host immune cells can be used as a biomarker in predicting cancer progression [61]. Furthermore, given the possible association between circulating microRNA and cancer immunity, studies on circulating microRNA are expected to lead to the future development of new therapeutic agents through immunomodulation. MicroRNAs represent a new source of reliable biomarkers that can be diagnostic, prognostic, and predictive during therapy of cancer patients and has been widely studied in prostate [62], renal [63], and urothelial carcinoma [64]. MicroRNA can be quantified by reverse transcription-PCR (RT-PCR), Northern blotting, in situ hybridization, gene expression microarray, or NGS technology but also with commercially available isolation kits including the miRNeasy Mini kit (Qiagen) [65], ZR urine RNA isolation kit (Zymo Research) [66] for bladder cancer; Acid phenol–chloroform plus Silica columns (BioSilica Ltd.) [67], Urine Exfoliated Cell and Bacteria RNA Purification Kit (Norgen) [68] for prostate cancer; TRIZOL reagent (Invitrogen) [69] and miRNeasy Serum/Plasma kit (Qiagen) [70] for gastric cancer.

#### 2.2.2. Long Non-Coding RNAs (lncRNAs)

Long non-coding RNAs (lncRNAs) are transcripts with length greater than 200 nucleotides encoding no protein and are gene regulators involved in many biological functions and dysregulated in various cancers [71]. Expression of lncRNAs is associated with a broad range of cellular processes, such as cell growth, survival, migration, invasion, and differentiation [72]. More recently, studies have investigated their possible role as biomarkers in cancer by highlighting the role of lncRNAs in carcinogenesis through impairment of cell cycle arrest and apoptosis [73]. Many lncRNAs are exosome-derived in urine and have been found to be more protected by RNAse activity. The gold standard method for lncRNA detection is quantitative RT-PCR [74]. Prostate cancer antigen 3 (PCA3) was the first lncRNA identified in 1999 mapped on chromosome 9q21–22 and found to be overexpressed in greater than 95% of prostate cancers [75]. The human urothelial carcinoma–associated 1 (UCA1), a 2314-bp lncRNA located on human chromosome 19, has been found to be upregulated in many cancers, such as hepatocellular cancer [76], colorectal cancer [77], gastric cancer [78], esophageal squamous cell carcinoma [79], and epithelial ovarian cancer [80].

#### 2.2.3. Other Urinary Small Non-Coding RNAs (sncRNAs)

Small non-coding RNAs are usually shorter in length by about 18–200 nucleotides. While mRNAs are highly susceptible to nucleases, sncRNAs, which are smaller in size, form stable complexes in urine, making them more resistant to nuclease [81]. They include small nuclear RNA (snRNA), small nucleolar RNA (snoRNA), Piwi-interacting RNA (piRNA) and tRNA-derived small RNA (tsRNA). They have diverse roles, which in conjunction with other molecules involve gene regulation through RNA interference or RNA modification. SncRNAs circulate as part of nucleoprotein complexes or membrane-coated microparticles such as exosomes [82]. Their role as a biomarker of cancers remains unclear [83].

## 3. Utility of Urine for Liquid Biopsy

While urine is a relatively cell-free biofluid, it contains large numbers of complex substances, including protein, circulating NAs (DNAs and RNAs), and extracellular vesicles (EVs). Since the yield and sensitivity of urine cfDNAs are comparable to blood cfDNAs, attention has been directed to urine sampling as an alternative body fluid source in lieu of blood to monitor clinical course and follow-up therapeutic effects [84]. Genomic abnormalities detected from urine NAs are shown to be useful in both urologic and non-urologic cancers. It has been shown that the sensitivity of cfDNA/ctDNA in urine is comparable to blood among patients with multiple cancers, such as urothelial carcinoma [85], breast cancer [84], colon cancer [41], and lung cancer [37]. One of the major advantages of using urine is its non-invasive nature of collection compared to tissue or blood, especially in patients requiring repeated sampling to monitor cancer progression and/or therapeutic effects [86]. Urine can be collected in large quantities, which solves one of the major problems with tissue biopsy or other liquid biopsy materials that often suffer from a limited number of samples. It is more patient-friendly since the collection of urine can be done anywhere as opposed to access to other body fluid or tissue which needs to be done in clinics or hospitals. Even in clinic settings, obtaining sufficient blood draws can be a challenge in some populations including geriatric patients, intravenous drug abusers, or anyone with thin veins [87]. Sampling cerebrospinal fluid (CSF) or gastric juice is even more invasive, and sophisticated techniques are required for their collection. Therefore, liquid biopsy using urine is expected to significantly reduce labor and cost as well as patients’ pain. Due to these advantages, urine liquid biopsy has been investigated for cancer screening, monitoring of cancer progression or recurrence, and the efficacy of chemo and radiation therapy.

### 3.1. Urinary Liquid Biopsy for Urological Cancers

Most of the studies regarding urine liquid biopsy have been performed on urological cancers, since many of the substances secreted from urological cancer are likely to drain directly into the urinary tract [27,88]. First morning urine contains the highest number of cells and cellular debris from the urological tract exfoliated in urine at night [89]. Both low-molecular-weight DNA (<100 bp) and high-molecular-weight DNA (≥1 kbp) can be detected in urine [84]. Urinary protein biomarkers for early detection of prostate cancer and bladder cancer have already been established and approved by the FDA, such as Nuclear Matrix Protein 22 (NMP22), Urovysion Fluorescence In Situ Hybridization (FISH), and Prostate Cancer gene 3 (PCA3) [90]. As a matter of fact, several tests based on urine liquid biopsy have been already included in the National Comprehensive Cancer Network (NCCN) Guidelines for Prostate Cancer Early Detection since 2020. These tests are Mi-Prostate scores that include measurements of PCA3 and TMPRSS2:ERG fusion gene expression in the urine, IntelliScore and SelectMDx, which may reduce the number of unnecessary biopsies [91]. In addition to ctDNA/cfDNA, the other NAs, such as mRNA [44,92], lncRNA [93], microRNA, piRNA [94], and circRNA [95], have been reported to be useful as biomarkers in urological cancers. The first commercial exosome-based prostate Intelliscore test for prostate cancer became available in 2016 [96]. Several urinary lncRNAs, such as FR0348383, MALAT1, and DD3 (PCA3), have been reported as better biomarkers in prostate cancer compared to serum prostate-specific antigens (PSA) [97,98]. Given its quality and accuracy, detection of urinary PCA3 has been approved by the FDA as a diagnostic tool for prostate cancer [98]. PCA3 levels have also been associated with tumor volume burden and extracapsular extension and provide prognostic information before a radical prostatectomy [99]. Urothelial carcinoma associated 1 (UCA1) is one of the most well studied genes in bladder cancer, and urinary lncRNA of UCA1 was often detected in patients with bladder cancer [100,101]. Currently, there are two clinical trials evaluating urine as a source for liquid biopsy. NCT04432909 is a prospective multi-center, single-blinded study to evaluate the utility of UroCAD for urothelial carcinoma diagnosis and follow-up in 500 participants (https://clinicaltrials.gov/ct2/show/NCT04432909, accessed on 19 May 2021). Patients with urothelial carcinoma prior to resection are compared with the patients being treated for other diseases but without any tumor to determine the sensitivity and specificity of UroCAD analysis, which will be compared with cytology and FISH. Another trial was reported at the American Society of Clinical Oncology Genitourinary (ASCO-GU) 2021 meeting by Zhang et al. from Shanghai, China, which is a prospective clinical trial that compares blood and urine liquid biopsy using PredicineCARE NGS 152 gene assay with the gold standard of tissue biopsy in 59 treatment-naïve bladder cancer patients. The mutation profiles of urine samples (sensitivity of 86.7%) were found to be very similar with tissue biopsy compared to blood liquid biopsy samples (sensitivity of 10.3%). At this point, we were unable to identify any current ongoing clinical trials in non-urological cancers. With increasing evidence, it is conceivable that detection of not only DNA and miRNA but also oncogenic lncRNAs in urine might enable early cancer diagnosis and can be promising therapeutic targets for patients with genitourinary cancer.

### 3.2. Urinary Liquid Biopsy for Non-Urological Cancers

There are numerous studies identifying common mutations in each type of cancer, such as EGFR mutation in lung cancer, that guide us in assigning a cell of origin to a biomarker like cfDNA. We have summarized these molecules detected in urine and the cancer type in Table 2. In addition, given the strength of liquid biopsy in longitudinal follow-up, we may discover a unique/novel biomarker for a particular patient of a particular cancer type that may become a strategy in the future. It is speculated that urinary RNAs may be also associated with clinical outcomes in patients with various types of cancers [102,103].

#### 3.2.1. Urine Liquid Biopsy in Lung Cancers

Conventional tissue biopsies are particularly cumbersome and carry potential risk for significant morbidity to lung cancer patients since they can cause pneumothorax and significant bleeding within the airway. Various urine liquid biopsy components have been investigated for patients with non-small cell lung cancer (NSCLC) and have been reported to reduce costs by improving detection of EGFR T790M mutations and reducing the complications associated with tissue biopsy [109]. Reckamp et al. studied 63 patients with advanced EGFR-mutant NSCLC and found that the sensitivities of tissue, plasma, and urine were 73%, 82%, and 75%, respectively, for T790M detection in these complementary specimens [36]. They also found a significant decrease in T790M MAF in urine in patients treated with Rociletinib (an EGFR tyrosine kinase inhibitor (TKI)), highlighting a potential of using urine for follow-up. These findings were confirmed in another study by Husain et al. who found that early kinetics of ctDNA in the urine of eight patients treated with Osimertinib, a third generation anti-EGFR TKI, correlated with tumor response [104]. These studies demonstrate that urine testing successfully identifies EGFR mutations in patients with advanced stage/metastatic NSCLC and has high concordance with tumor tissue and plasma and can be used as a viable approach for assessing EGFR mutation status. In advanced NSCLC, Wu et al. demonstrated a good correlation and complementarity between genomic profiles of cfDNA extracted from plasma, sputum, and urine compared to tissue [105]. In early-stage NSCLC, the analysis of DNA methylation at cancer-specific loci in urine were shown to help characterize nodules after screening via computed tomography (CT) [106]. Thus, various studies have demonstrated the utility of urine liquid biopsy not only as a diagnostic but also a prognostic and predictive marker in NSCLC.

#### 3.2.2. Urine Liquid Biopsy in Breast, Gynecological, and Gastrointestinal Cancers

Some have investigated the role of urine liquid biopsy in early breast cancer. In a prospective study, Zhang et al. compared serum and urine ctDNA levels using a droplet digital PCR (ddPCR) technique of 200 breast cancer patients and healthy volunteers [107]. The authors found 3.5-fold higher levels of ctDNA as well as wild-type PIK3CA genotype in early breast cancer patients compared to healthy volunteers. These results demonstrate that urinary ctDNA is capable of discerning between healthy populations while providing early disease detection, especially in high-risk individuals. Zhang et al. also evaluated a decline of urinary ctDNA following initial treatment and found a 6.8-fold decrease [107].

Among the tested 10 microRNAs, miR-10b-5p was identified as a candidate biomarker for endometrial and ovarian cancer [108]. It was found to be elevated in patients with endometrial cancer compared to healthy women; however, its relevance in ovarian cancer remains unelucidated. MiR-200c-3p was found to be enriched in the urine of endometrial cancer patients, paving the way for the development of a non-invasive biomarker for early detection [110]. Abnormal lncRNA UCA1 expression has been linked to adverse clinicopathological characteristics including lymph node metastasis, chemoresistance, and poor overall survival in both cancers [80,111].

Identification of biomarkers for gastric cancer still remains a challenge. The most frequently used tumor markers include CEA, CA19-9, CA72-4, CA50, pepsinogen, and alfa fetoprotein, however their sensitivity and specificity are poor and hence not specific to a diagnosis of gastric cancer. Hung et al. reported that miR-376c promotes gastric cancer cell proliferation and migration, and it was increased in urine and plasma of gastric cancer patients [112]. Kao et al. detected miR-21-5p levels in the urine of gastric cancer patients pre- and post-op at one and three months and found that its levels consistently decreased following gastric surgery [69]. MiR-6807-5p and miR-6856-5p were also found to be significantly increased in the urine of gastric cancer patients but fell to almost non-detectable levels following gastric resection [70]. These results appear promising for both early detection and prognosis of patients with gastric cancer.

KRAS mutations were detected from the cfDNAs in the urine of advanced colorectal cancer patients. This was the first reported urinary cfDNA as a biomarker in a non-urological cancer, proving that the kidney barrier in humans is permeable to DNA molecules large enough to be analyzed by standard genetic technologies [113]. Su YH et al. compared the concentration of DNA in different body fluids and found that it was similar in urine compared to serum, but it was significantly lower in plasma than in either urine or serum (p < 0.05). They also reported that when DNA was derived from 10 μL of body fluid in each mutation assay, the mutated KRAS DNA detection was comparable among serum, plasma, and urine. However, in patients with colorectal cancer, when a larger amount of body fluid (200 μL) was used the detection rate of the KRAS gene in urine was significantly higher (95%) than in serum (35%) or plasma (40%). These findings suggest that inhibitory factors (such as DNase) in serum and plasma might be less abundant in urine, and that urine does not usually contain large molecules, such as protein, that can interfere with PCR amplification compared to blood or serum, as they are filtered by the kidneys [41].

## 4. Limitations of Urinary Liquid Biopsy

By definition, urine is generated by the kidney and there are many components that may not get filtered in the urine compared to blood. Therefore, urine liquid biopsy has been more intensively studied in genitourinary cancers and is one of the major limitations in non-urological cancers. Since urine is a dynamic body fluid, concentrations change with hydration status, renal pathology, urine volume, and effect of medications. Hence, concentrations will most likely not be reliable with a high degree of variability within the urine composition and will require an absolute amount or centrifugation. Therefore, measuring 24 h urine volumes would be the gold standard to assess hydration status. Measuring creatinine ratios or specific gravity remain as other possibilities and potentially more feasible alternatives. Despite being a useful tool for diagnosis, prognosis, and a predictive marker for treatment response, a major limitation of urine cfDNA-based tests includes lack of specificity. Increased levels of cfDNA are seen in non-malignant conditions such as trauma, inflammation, pregnancy, autoimmune conditions like lupus, and infections such as tuberculosis. Due to these very reasons, cfDNA-based tests lack application in the clinical setting [36]. Mutation rates in individuals may be influenced by environmental and physiological factors [114], and spontaneous mutations known to typically contribute to cancer development can occur with increasing age but may not directly cause cancer. There is also less abundance of mRNA in urine, a lack of stable targeted molecules, along with possible contamination of cellular RNA during sample preparation [115]. Thus, utilization of urine liquid biopsy in pre-symptomatic stages may yield false positive results and overdiagnosis of cancer. With regards to methodology, microchip analysis is an efficient method to screen for urine biomarkers; however, challenges when applying this method include repetitive sequences in the discovery phase miRNAs when designing probes or primers (due to short length of nucleotides), which may result in artifacts [108] masking the results of microchip analysis. Varied analytical methods include NGS, RT-PCR, and microarray, which can lead to aberrational findings [116]. Variations exist in assay protocols and sample handling despite the same analytical method performed. More importantly, why certain specific RNA extraction kits are used for detection of biomarkers in different studies depending on the cancer site, remains elusive. Lack of large multicenter studies remain the major reason for precluding its adoption in clinical practice.

While there are several limitations to urine as liquid biopsy, it can also be used to our advantage. The biggest advantage of urine is that an unlimited amount can be collected. Instead of an absolute value, urine samples can be collected as a set quantity per day and quantified as a fraction of the total quantity (especially in patients suffering from excessive diuresis). Ideally, it would be beneficial to confirm the presence of sufficient amounts and quality of ctDNA to identify the most appropriate ctDNA quantification methods to maintain uniformity and improve the sensitivity of ctDNA detection to anticipate drug resistances by urine biopsy. In addition to looking into ctDNA, we can look into smaller nucleic acids such as messenger RNA, micro-RNA, circular RNA, transfer RNA, or even RNA in exosomes. Analyzing exosomes in the future can become an important strategy as cells communicate through exosomes. More recently, with newer tools like SiRe NGS panel testing [12] or the TargetPlex FFPE Direct DNA library preparation kit [117] being applied to patient blood and tissue samples with advanced-stage NSCLC, we cannot help but speculate that these more cost-effective methods may gain more widespread application in urine liquid biopsies in the future. Considering the positive effects on biomarker studies and beyond, we hope that funding bodies will take steps to complement the current emphasis on these novel studies and support programs for reproduction studies of existing findings to validate their clinical utility.

## 5. Conclusions and Future Perspectives

Changes in genomic and genetic material in the urine potentially precede changes in imaging and can detect minimal tumor burden of urological and non-urological cancers. There still remains a need for standardized methods and normalization procedures. Despite the non-invasive nature of sample collection and its potential benefits, this newer urine-based approach still requires large-scale research for validation by large cohorts prospectively. Although a promising innovation, an important question that remains to be answered is whether urine biomarkers offer better profiling for disease recurrence and whether urine biomarker elevation-driven interventions translate into better outcomes.

## Figures and Tables

**Table 1:** Advantages and disadvantages of standard tissue biopsy versus blood liquid biopsy versus urine liquid biopsy.

	Standard Tissue Biopsy	Blood Liquid Biopsy	Urine Liquid Biopsy
Components	Cell structure, grade, stromal and immune cells, Lymphovascular invasion, DNA seq, RNA seq, gene signatures	CTCs, cell free nucleic acids, exosomes, tumor educated platelets	Cell free DNA, urinary mRNA, miRNA, lnc RNA, other snc RNA, exosomes
Advantages	Standard of care Standard technique, low FNR Histological information and immunohistochemical profiling excellent	Minimally invasive procedure Early detection and molecular profile assessment Intratumor heterogeneity Real time monitoring of cancer evolution Corelates with tumor burden Identifies genetic markers of treatment and treatment resistance DNA fresh and not modified by storage technique Quick turnaround testing time for ctDNA ctDNA more beneficial in metastatic setting	Noninvasive procedure Early detection and molecular profile assessment Intratumor heterogeneity Large quantities available and centrifuged for concentrates High DNA yield Identifies genetic markers of treatment and treatment resistance Good for longitudinal follow up ucfDNA can potentially help in localizing “cancer of unknown primary”
Disadvantages	Invasive procedure, involves patient risk Lacks assessment of intratumor heterogeneity Time period of analysis fixed Repetitive invasive biopsies cumbersome Early detection of cancer not possible DNA quality highly variable in FFPE Variable quantity of DNA based on sampling methods, high risk of DNA degradation	Investigational setting High FNR Lack of standardized technique for cfDNA and cellular genomic DNA ctDNA quality and extraction methods. Short half life of CTCs (1–2.4 h) in peripheral blood	Investigational setting No histological assessment Effect of hydration status and medications ucfDNA integrity sensitivity and specificity issues Artifacts from microchip analysis Variations in assay protocols/sample handling Measurement of urinary RNAs challenging Lack of large multicenter studies

CTC: Circulating tumor cells; FNR: False negative rate; lnc RNA: Long non-coding RNA, sncRNA: small non-coding RNA; ucf DNA: urine cell-free DNA; RT-PCR: Reverse transcription polymerase chain reaction; FFPE: Formalin fixed paraffin embedded; ctDNA: circulating tumor DNA.

**Table 2:** Application of urine liquid biopsy in non-urological cancers.

Study, Reference Number	Cancer Type	Early Stage, Advanced or Metastatic	No of Patients	Molecules Assessed	Methodology/Quantitative Analysis	Clinical Application of Urine Biopsy	Sensitivity in Urine
Reckamp [37]	NSCLC	Advanced Stage	63	ctDNA for EGFR T790M mutation	ddPCR, NGS	Predictive response to Rociletinib (EGFR TKI)	75%
Husain [104]	NSCLC	Advanced Stage	8	ctDNA for EGFR T790M mutation	ddPCR, NGS	Predictive response to Osimertinib (III generation EGFR TKI)	86%
Wu [105]	NSCLC	Advanced Stage & Metastatic	50	TP53 and EGFR mutation	PCR, NGS	Detection of driver gene alterations	60%
Liu [106]	NSCLC	Early stage	74	DNA methylation	Methylation specific PCR	Early detection after incidental finding of nodule on CT chest	73%
Zhang [107]	Breast	Early stage	200	ctDNA for PIK3CA	ddPCR	Prognostic and predictive	77%
Ritter [108]	Endometrial & Ovarian	Early stage	10	MiR-10b-5p	RT-qPCR, Human miRNA V21.0 microarray	Early detection	50%
Kao [69]	Gastric	Early stage	50	MiR-21-5p	Quantitative stem loop RT- PCR	Predictive	NA
Iwasaki [70]	Gastric	Early stage	197	MiR-6807-5p MiR-6856-5p	miRNeasy kit (Qiagen), miRNA microarray	Early detection and Prognostic	63.4%
Su [41]	Colorectal	Advanced stage	20	cfDNA KRAS mutation	RT-PCR	Early detection	95%

NSCLC: Non-small cell lung cancer; ddPCR: Droplet digital polymerase chain reaction; NGS: Next generation sequencing; RT-PCR: Reverse transcription polymerase chain reaction; ctDNA: circulating tumor DNA.
